# GDF3 Protects Mice against Sepsis-Induced Acute Lung Injury by Suppression of Macrophage Pyroptosis

**DOI:** 10.3390/ph17030268

**Published:** 2024-02-20

**Authors:** Jiaxi Lei, Lu Wang, Lijuan Zou, Huijuan Wang, Yunlong Zhang, Shiping Liu, Mingliang Pan, Xue Zhu, Liying Zhan

**Affiliations:** Department of Critical Care Medicine, Renmin Hospital of Wuhan University, Wuhan 430060, China; 2021283020163@whu.edu.cn (J.L.); wanglu@whu.edu.cn (L.W.); 15926366005@139.com (L.Z.); 2021283020268@whu.edu.cn (H.W.); 2014302180108@whu.edu.cn (Y.Z.); liushiping@whu.edu.cn (S.L.); 2023203020045@whu.edu.cn (M.P.); 2023283020118@whu.edu.cn (X.Z.)

**Keywords:** sepsis, acute lung injury, growth differentiation factor 3, pyroptosis, macrophages

## Abstract

Sepsis-induced ALI is marked by physiological, pathological, and biochemical irregularities caused by infection. Growth differentiation factor 3 (GDF3) is closely associated with the inflammatory response. Accumulating evidence has demonstrated a close relationship between GDF3 expression and the severity and prognosis of sepsis. However, the precise mechanism by which GDF3 protects against ALI induced by sepsis is still unclear. Following the intravenous administration of GDF3 in this research, we noted a rise in the survival rate, a decrease in the severity of histopathological damage as evaluated through HE staining, a decline in the count of inflammatory cells in bronchoalveolar lavage fluid (BALF), a reduction in the ratio of lung wet/dry (W/D) weight, and a noteworthy decrease in the levels of pro-inflammatory cytokines in both serum and BALF when compared to septic mice who underwent cecal ligation and puncture (CLP). These collective findings unequivocally indicate the protective effects of GDF3 against sepsis-induced ALI. In addition, the GDF3 group showed a significant reduction in the mRNA expression of Caspase-1 and NLRP3 when compared to the CLP group. Following this, we performed in vitro tests to confirm these discoveries and obtained comparable outcomes, wherein the administration of GDF3 notably decreased the levels of Caspase-1 and NLRP3 mRNA and protein in macrophages in comparison to the LPS group. Furthermore, GDF3 exhibited the capacity to reduce the secretion of inflammatory molecules from macrophages. By illuminating the mechanism by which GDF 3 regulates macrophages, this offers a theoretical basis for preventing and treating sepsis-induced ALI.

## 1. Introduction

Sepsis, resulting from a dysregulated host response to infection, it poses a life-threatening risk to organ function [1] and a significant public health challenge [2]. During sepsis, the lungs are particularly susceptible to injury, with over 50% of patients experiencing acute lung injury (ALI) [3]. There is a significant prevalence and severity of acute respiratory distress syndrome (ARDS) in patients that frequently accompanies sepsis. Emerging evidence highlights the important role of immunometabolism in the inflammatory response and development of ALI [4]. As the most common immune cells in the lungs at homeostasis, macrophages play an essential role in the development of sepsis-induced ALI [5]. However, the pathogenesis and pathophysiology of macrophage involvement in sepsis-induced ALI are still poorly understood.

Pyroptosis is a programmed cell death characterized by inflammation [6]. In contrast to apoptosis and autophagy, it is a distinct form of cell death. Cysteine-aspartic protease 1 (caspase-1) plays an important role in pyroptosis, which is characterized by the activation of the NLRP3 inflammasome and the release of numerous pro-inflammatory factors [7]. There is growing evidence that macrophage pyroptosis plays a significant role in the development of multiple organ damage during sepsis [8]. Pyroptosis of macrophages can occur in the early stages of infection, rapidly initiating an immune response by releasing inflammatory factors. Immune cells can then recognize and eliminate intracellular bacteria through the lysis of these pyroptotic cells [9]. Based on initial findings, targeting the inhibition of NLRP3-mediated pyroptosis could potentially be a crucial approach for alleviating ALI [10]. Using pyroptosis inhibition as a clinical prevention or treatment for ALI and ARDS may hold promise in the future.

A member of the TGF-β superfamily, growth differentiation factor 3 (GDF3), also known as Vgr-2, was initially discovered for its role in regulating early embryonic development [11]. GDF3 is expressed in various tissues including the embryonic skeleton, adult bone marrow, spleen, and fat. It plays a role in regulating embryonic development and differentiation, as well as being involved in adipose tissue metabolism, energy metabolism, inflammatory response, and stress response [12,13,14]. Macrophage functions are believed to be modulated by GDF3 in recent studies [14]. However, there is still much to learn about how GDF3 inhibits NLRP3 inflammasome-dependent pyroptosis in the context of ALI, which is worthy of further study. Hence, the purpose of this study was to investigate GDF3’s effect on ALI induced by cecal ligation and puncture (CLP).

In this study, we established mouse models of CLP to investigate the relationship between pyroptosis and GDF3. We conjecture that GDF3 can potentially inhibit pyroptosis by inhibiting the NLRP3/Caspase-1 signaling pathway, thereby mitigating damage to lung tissue. Ultimately, we discovered that GDF3 may be involved in safeguarding against lung injury by activating the NLRP3/Caspase-1 signaling pathway and suppressing cell pyroptosis in CLP mice.

## 2. Results

### 2.1. GDF3 Protects Mice against CLP-Induced ALI

**Figure 1:**
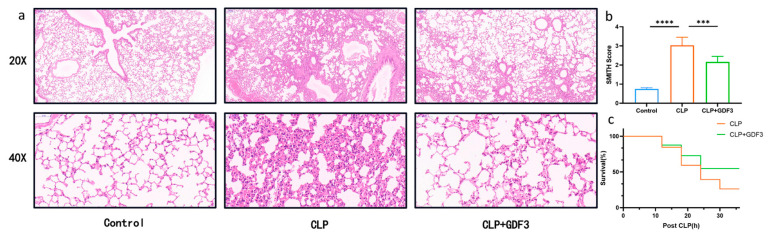
GDF3 protects mice against CLP-Induced ALI. (**a**) Representative images of lung tissue pathology were obtained by performing H&E staining on each group of mice. (**b**) Histological scoring was conducted to assess the injury of CLP-induced ALI. (**c**) A Kaplan–Meier survival curve was generated for each group of mice to compare mortality rates (*n* = 10). Values are means ± SEM. *** *p* < 0.001, **** *p* < 0.0001.

To examine the protective effects of GDF3 against CLP-induced ALI, an H&E staining of lung tissue was performed to examine its histology. In comparison to the sham group, the CLP group showed increased bleeding, lung edema, thickened alveolar walls, and neutrophil infiltration. Nevertheless, these histopathological changes were mitigated through GDF3 treatment (Figure 1a). Furthermore, GDF3 treatment reduced the histological lung injury score (Figure 1b). It is noteworthy that mice treated with GDF3 exhibited a significantly higher survival rate within 36 h after CLP when compared to those treated with CLP alone (Figure 1c). These findings strongly suggest that GDF3 treatment effectively alleviates CLP-induced ALI.

### 2.2. GDF3 Alleviated the Increased Alveolar–Capillary Permeability in Lung Tissue

**Figure 2:**
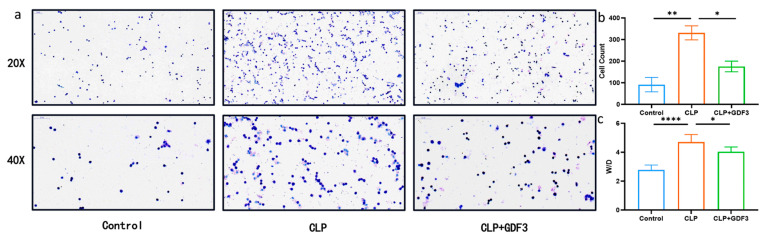
GDF3 alleviated the increased alveolar–capillary permeability in lung tissue. (**a**) Cells in mouse BALF samples were examined using Wright’s Giemsa staining. (**b**) The total cell counts in BALF samples were measured in CLP-induced ALI mouse models treated with or without GDF3 (*n* = 6). (**c**) The W/D ratios of the lung were quantified in mice treated with GDF3 24 h after CLP (*n* = 6). Values are means ± SEM. * *p* < 0.05, ** *p* < 0.01, **** *p* < 0.0001.

Excessive infiltration of inflammatory cells is closely associated with the pathogenesis of ALI [15]; increased permeability of the alveolar capillaries plays a crucial role in this process [16]. In this study, Wright’s Giemsa staining was used to examine the potential effects of GDF3 on inflammatory cell infiltration into lung tissue 24 h after CLP-induced ALI (Figure 2a). Analysis of BALF showed that neutrophils were the predominant inflammatory cells infiltrating the lung tissue, and their numbers decreased after pretreatment with GDF3 (Figure 2b). Additionally, mice treated with GDF3 exhibited a lower ratio of W/D compared to mice subjected to CLP (Figure 2c). These results demonstrate that GDF3 could alleviate the increased alveolar–capillary permeability in lung tissue.

### 2.3. GDF3 Inhibits the Levels of Cytokines in CLP-Induced ALI

**Figure 3:**
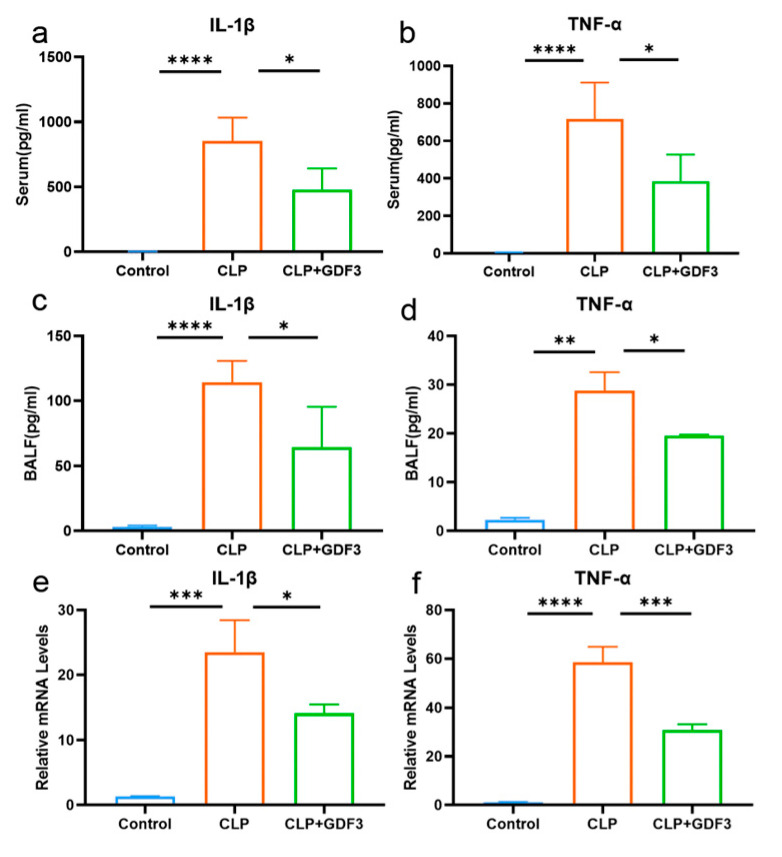
GDF3 inhibits the levels of cytokines in CLP-induced ALI. (**a**,**b**) Levels of IL-1β, TNF-α in serum (*n* = 6). (**c**,**d**) Levels of IL-1β, TNF-α in BALF (*n* = 6). (**e**,**f**) The mRNA expression levels of IL-1β and TNF-α were assessed in lung tissues. Part of the above cytokines were measured by multiplex secretome analysis (*n* = 6). Three independent experiments were conducted. All values are means ± SEM. * *p* < 0.05, ** *p* < 0.01, *** *p* < 0.001, **** *p* < 0.0001.

We measured the levels of cytokines in mice’s serum and bronchoalveolar lavage fluid (BALF), including IL-1β and TNF-α (Figure 3a–d). The CLP group exhibited a notable rise in serum levels of IL-1β and TNF-α when compared to the control group. However, after treatment with GDF3, the levels of these cytokines decreased. Similar results were found in BALF. Additionally, the expression of cytokine mRNA in mouse lung tissue also showed consistent results (Figure 3e,f). These findings provide evidence suggesting that GDF3 exhibits anti-inflammatory effects in mice with CLP-induced acute lung injury (ALI).

### 2.4. GDF3 Reduces Pyroptosis in CLP-Induced ALI

**Figure 4:**
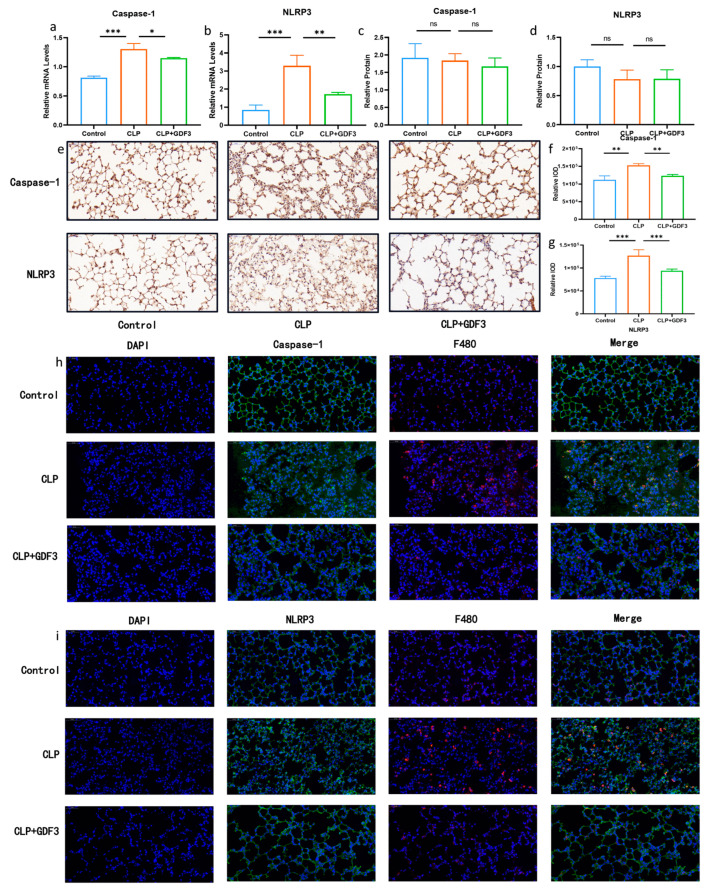
GDF3 reduces pyroptosis in CLP-induced ALI. (**a**,**b**) Caspase-1 and NLRP3 mRNAs were assessed by RT-qPCR (normalized to β-Actin) (*n* = 6). (**c**,**d**) Western blot analysis of Caspase-1 and NLRP3 in the lungs collected from ALI induced by CLP. (**e**) Immunohistochemistry of Caspase-1 and NLRP3 expression in the lung tissues of each group of mice (magnification ×400). (**f**,**g**) Semi-quantitative analysis of Caspase-1 and NLRP3 expression of lung tissues based on the immunohistochemistry. (**h**,**i**) The images presented above represent immunofluorescence evidence of Caspase-1 and NLRP3 expression. Blue DAPI staining is applied to the nuclei, F4/80 is stained in red, and Caspase-1 and NLRP3 are stained in green by specific antibodies. Values are means ± SEM. * *p* < 0.05, ** *p* < 0.01, *** *p* < 0.001.

Western blotting and RT-qPCR were used to detect the levels of NLRP3 and Caspase-1 in mouse lung tissue, two markers of pyroptosis. It was found that the relative mRNA levels in the lung tissue of the CLP (cecal ligation and puncture) group were significantly higher than that of the control group, indicating the occurrence of cell pyroptosis (Figure 4a,b). Notably, GDF3 treatment resulted in a decrease in the mRNA levels of pyroptosis markers, indicating its potential to attenuate pyroptosis in the lungs of CLP mice. However, the observed change was not substantial regarding the protein level (Figure 4c,d). We also examined the expression of pyroptosis-related indicators (NLRP3 and Caspase-1) in the lung tissues of each group of mice using immunohistochemistry. The representative images and semi-quantitative analysis demonstrated an increase in the expression of pyroptosis-related indicators in the CLP group, which decreased after GDF3 treatment (Figure 4e–g). Pyroptotic cells were detected in vivo using an antibody specific for macrophages, F4/80, and biomarkers indicative of pyroptotic activity (Figure 4h,i). It is worth noting that upregulated expression of pyroptotic molecules NLRP3 and Caspase-1 was observed, and this upregulation was effectively suppressed upon GDF3 treatment.

### 2.5. GDF3 Inhibited LPS-Induced Pyroptosis in Macrophages

**Figure 5:**
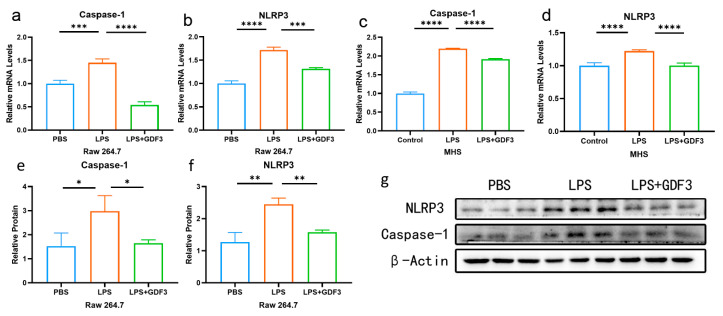
GDF3 inhibited LPS-induced pyroptosis in macrophages. (**a**–**d**) A RT-qPCR was used to measure Caspase-1 and NLRP3 relative mRNA expression levels (normalized to β-Actin) in different macrophages. (**e**–**g**) Protein expression levels of Caspase-1 and NLRP3 were assessed using Western blotting. Three independent experiments were conducted to obtain the results. Values are means ± SEM. * *p* < 0.05, ** *p* < 0.01, *** *p* < 0.001, **** *p* < 0.0001.

There was a significant increase in mRNA levels for Caspase-1 and NLRP3 in the LPS group compared to the PBS group. Conversely, the protein levels of Caspase-1 and NLRP3 showed a significant decrease after GDF3 treatment (Figure 5a–d). Similar trends were observed for the protein levels of the pyroptosis marker (Figure 5e–g). These findings suggest that GDF3 can impact pyroptosis in LPS-induced macrophages, including Raw264.7 and MHS cells.

### 2.6. GDF3 Reduced the Levels of Cytokines in LPS-Induced Macrophages

**Figure 6:**
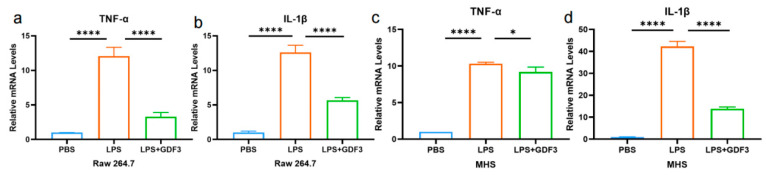
GDF3 reduced the levels of cytokines in LPS-induced macrophages. (**a**–**d**) TNF-α and IL-1β mRNAs were assessed by RT-qPCR (normalized to β-Actin) in macrophages (Raw264.7 and MHS cells). Values are means ± SEM. * *p* < 0.05, **** *p* < 0.0001.

GDF3 significantly reduced the levels of pro-inflammatory cytokines IL-1β and TNF-α released from inflammatory cells in mice (Figure 3). This is supported by qPCR analysis of the macrophage cell line RAW264.7, which indicated a significant increase in the mRNA expression of TNF-α and IL-1β in LPS-treated cells. However, treatment with GDF3 counteracted this increase (Figure 6a,b). Additionally, we observed a protective effect of GDF3 on inflammatory damage in MHS cells (Figure 6c,d). Overall, these results demonstrate that GDF3 inhibits LPS-induced infiltration of inflammation cells, thereby reducing the production of pro-inflammatory cytokines in ALI.

## 3. Discussion

Among the various complications associated with sepsis, pulmonary injury is particularly severe [17]. Acute lung injury (ALI) is a clinical syndrome marked by an excessive inflammatory response, increased pulmonary capillary permeability, and severe hypoxemia [18]. Acute respiratory distress syndrome (ARDS), which has a high mortality rate, can result from ALI in severe cases [19]. The etiology of ALI/ARDS is multifactorial, including causes such as sepsis, pancreatitis, trauma, pneumonia, and aspiration [20]. Among these, infection is the most common cause.

GDF3 is expressed in various tissues including embryonic skeletal tissue, adult bone marrow, spleen, and fat [11]. Its role spans across multiple processes such as the regulation of embryonic development, differentiation, adipose tissue metabolism, energy metabolism, inflammatory response, and stress response [21,22,23]. This is achieved through interactions with ALK4/7 on the cell membrane and involvement in the TGF-β/Smad classical signaling pathway [11]. Additionally, Varga et al. discovered that GDF3 regulates muscle regeneration and enhances the fusion of primary myoblasts [24]. In white adipose tissue, insulin–GDF3–ALK7 plays an important role in regulating both physiological and pathological fat accumulation [25]. Recently, a novel interactive mechanism linking Gdf3 to ALK7 regulates adipocyte–macrophage communication to regulate adipogenesis [26]. Furthermore, the effects of GDF3 on muscle repair are believed to compensate for the age-related decline in reparative macrophages [13]. Previous investigations conducted by our team have confirmed a significant upregulation of GDF3 expression in the serum of sepsis patients in comparison with healthy controls. Furthermore, this increase in expression has been closely associated with disease severity and prognosis. In a mouse sepsis model, administration of GDF3 has demonstrated improvements in cardiac function, suppression of the inflammatory cytokine IL-1β, and a reduction in mortality [27]. However, the precise mechanism of GDF3 in acute lung injury (ALI) remains incompletely understood. As a result of the experiments, lung tissues in the model group had a higher wet-to-dry weight ratio, accompanied by substantial neutrophil infiltration, alveolar collapse, macrophage necrosis, and elevated levels of TNF-α and IL-1β in serum and lavage fluid, thus validating the successful creation of the ALI mouse model. However, intervention with GDF3 significantly reduced the wet-to-dry weight ratio of lung tissues, improved lung tissue structure, and diminished inflammatory cell infiltration, and subsequent investigations displayed a noteworthy decrease in the levels of inflammatory cytokines in serum and lavage fluid. This suggests that GDF3 has the potential to effectively alleviate lung edema, ameliorate inflammatory damage in the lungs, and restrain the release of pro-inflammatory cytokines in ALI mice, ultimately exerting a therapeutic effect on ALI, which aligns with our previous results obtained from mouse models induced by LPS [27].

Pyroptosis is an inflammatory form of cell death primarily triggered by pathological conditions [28]. Inflammasomes trigger caspase-1 activation by binding to NLRP3 receptors, inducing GSDMD activation and inflammatory cytokine secretion within the compartment. The classical pathway is mediated by caspase-1, which is activated by NLRP3 receptors [29]. The swelling of cells, the rupture of cells, and the inflammatory response that follows are all caused by this process [30]. Pyroptosis plays a crucial role in the body’s immune response and is implicated in the development of tumors, infectious diseases, and atherosclerosis [31]. Increasing evidence suggests that macrophage pyroptosis is a significant mechanism underlying multi-organ damage in sepsis [32,33]. Therefore, it is hypothesized that GDF3 may be involved in the function of macrophages in sepsis-induced lung injury and that GDF3 can protect against sepsis-induced acute lung injury by inhibiting macrophage pyroptosis. HE staining, as well as immunohistochemistry of septic mice lung tissues, confirmed the presence of pyroptosis. It has been reported that GDF3 can enhance macrophage phagocytosis mediated by LXRα and improve survival in CLP-induced sepsis, exerting a protective effect in preventing acute lung injury [14]. Consistent with this, after GDF3 treatment, pyroptosis markers were significantly decreased in the lungs of septic mice. Furthermore, immunofluorescence staining demonstrated co-localization of the pyroptosis markers Caspase-1 and NLRP3 with the macrophage-specific antibody F4/80, providing further evidence that sepsis-induced lung injury can be prevented by inhibiting macrophage pyrolysis by GDF3. In in vitro experiments, treatment of Raw264.7 macrophages with GDF3 significantly reduced the protein and mRNA levels of Caspase-1 and NLRP3 compared to LPS-treated macrophages.

It is noteworthy that we did not observe significant differences in the protein levels of pyroptosis markers, Caspase-1 and NLRP3, in the lung tissues of the various groups of mice in the mouse CLP model. The limited representation of alveolar macrophages in lung tissue could potentially explain these findings [34]. Immunofluorescence results indicate that macrophage apoptosis primarily takes place within the cells, a finding supported by our in vitro experiments. As a result, it is conceivable that the presence of other cell types may have contributed to the absence of significant variations in apoptosis markers among the different groups of mice’s lung tissues. Future investigation should focus on elucidating the specific cell types involved and their respective underlying mechanisms.

The above research findings provide potential insights into the protective role of GDF3 in CLP-induced ALI. However, the investigation has only preliminarily focused on the NLRP3 inflammasome signaling pathway, with limited research on the relevance of other inflammasomes. Furthermore, the upstream regulation and underlying mechanisms of the NLRP3 inflammasome pathway have not been fully elucidated. Therefore, further experimental research is still needed to enrich and advance the theoretical foundations of ALI, with the goal of improving the prognosis of the disease.

## 4. Materials and Methods

### 4.1. Mice

Wild-type (WT) mice of the C57BL/6 strain weighing 22–25 g (8-week old, male) were obtained from the Experimental Animal Center of Wuhan University, China. An environment that was pathogen-free (SPF) for a minimum of one week was provided to the mice before the experiment, as well as ad libitum access to food and water for one week prior to the experiment. The Chinese Experimental Animal Administration Legislation required that all experimental procedures be conducted in strict accordance with its provisions and general recommendations. This was approved by the Laboratory Animal Ethics Committee of Remin Hospital of Wuhan University [WDRM NO.20230312B, Date: 2023-03-27].

### 4.2. Cell Culture

The China Center for Type Culture Collection (CCTCC) provided raw264.7 and MHS cells for this study. The cells were cultured under the same conditions, using Dulbecco’s modified Eagle’s medium (DMEM) (Basal Media Technologies, Shanghai, China) a 10% Fetal Bovine Serum (FBS) supplement (Procell Life Science & Technology Co., Ltd., Wuhan, China), 100 U/mL penicillin and 100 μg/mL streptomycin (Servicebio, Wuhan, China) are included. Incubation temperatures were 37 °C and 5% CO_2_ with the cells being stimulated with either PBS or LPS (10 mg/mL) for 24 h after reaching 60–70% confluency.

### 4.3. Mouse Model Preparation and Treatment

An induction of polymicrobial sepsis by CLP was performed in mice as described previously [14]. We injected recombinant mouse GDF3 protein (20 μg/kg body weight) (R&D Systems, MN, USA) or BSA vehicle into wild-type (WT) mice’s tail vein 3 h before CLP to determine whether it protects against polymicrobial sepsis in vivo. Then, pentobarbital (intraperitoneally, 50 mg/kg) was injected into mice to anesthetize them. The cecum was gently compressed to facilitate the release of fecal matter into the abdominal cavity. Subsequently, the cecum was anastomosed, and the consecutive layers of the abdomen were sutured. Following the surgical procedure, we administered pre-warmed buffered saline subcutaneously (0.05 mL/g body weight). It was not necessary to use antibiotics in the CLP model. In the 36 h following the CLP operation, the survival rate of each animal was monitored every six hours. After CLP surgery, mice were collected for blood and bronchoalveolar lavage fluid (BALF) collection 24 h later to assess cytokine levels.

### 4.4. Cell Count and Classification of Bronchoalveolar Lavage Fluid (BALF)

We opened the mouse chests and removed the lungs with the tracheotomy tube still in place. A ligation was performed on the left main bronchus and three lavages were performed on the right lung with 1.0 mL of PBS, resulting in approximately 0.8 mL of bronchoalveolar lavage fluid (BALF). Cell counting was performed using a Cell counter (Countess 3, Thermo Fisher,Waltham, MA, USA). The remaining BALF was then centrifuged (1500× *g*, 10 min, 4 °C) for Wright’s Giemsa staining, and the supernatant was used for analyzing cytokines

### 4.5. Lung Histology and Lung Injury Score

We fixed the upper portion of the lungs in paraformaldehyde 4%, embedded them in paraffin, and cut them into thin slices of 5 mm thickness. Staining with hematoxylin and eosin (H&E) was followed by scanning the tissue section with a digital scanner (Pannoramic MIDI, 3DHISTECH, Budapest, Hungary) for histopathological analysis.

We utilized a modified histological scoring system, known as the Smith Score [35], to evaluate lung injury. There are four levels of damage for tissue specimens: mild, moderate, severe, and maximal. The scoring system assessed the following parameters: (I) pulmonary edema, (II) infiltration of inflammatory cells in the alveolar and interstitial spaces, (III) alveolar and interstitial hemorrhage, and (IV) atelectasis and formation of the hyaline membrane. Scores ranging from 0 to 4 illustrated varying levels of lung involvement: mild (20–25%), moderate (25–50%), severe (50–75%), and very severe (>75%).

### 4.6. Tissue Wet-to-Dry Weight Ratio

In order to obtain the wet weight of the left lung, the lower portion was dissected and immediately weighed. After the dry weight of the lung tissue was obtained, it was weighed again using the dry weight obtained by drying it in an oven at 60 °C for approximately 48 h. As an indicator of lung edema, we calculated the wet-to-dry weight ratio (W/D).

### 4.7. Wright’s Giemsa Staining

The centrifuged cells were fixed in 4% paraformaldehyde and evenly spread onto slides. Wright’s Giemsa staining was performed, followed by histopathological scans using a tissue section digital scanner (Pannoramic MIDI, 3DHISTECH, Budapest, Hungary).

### 4.8. Cytokines of the Serum and the BALF

We separated the serum and the BALF using a centrifuge at 1500× *g* for ten minutes. IL-1β and TNF-α levels were measured by ABclonal Technology using a multiplex secretome analysis (ABplex Custom Panel Assay Kit).

### 4.9. Immunohistochemistry

The lung tissue underwent fixation in 4% paraformaldehyde for 48 h, followed by embedding in paraffin and cutting into 3 μm thick sections. Immunostaining was performed with specific antibodies targeting NLRP3 (1:100, Affinity, Liyang, China) and Caspase 1 (1:100, Affinity, Liyang, China). An immunohistochemistry kit (Servicebio, Wuhan, China) was utilized according to the manufacturer’s instructions. Immunohistochemistry slide scans were conducted using a tissue section digital scanner (Pannoramic MIDI, 3DHISTECH, Budapest, Hungary). The Image J 1.53k software (National Institutes of Health) was used to analyze protein expression levels.

### 4.10. Immunofluorescence Staining

Multiple targets can be labeled by utilizing various fluorescent labels. Through repeated immunolabeling and the utilization of different fluorescent tyramides, dual fluorescence staining can be performed. This is achieved using Tyramide Signal Amplification (TSA) technology for immunofluorescence staining. Paraffin sections were used for immunofluorescence staining. After being deparaffinized, antigen repair, sealing with hydrogen peroxide, and serum blocking, the sections were incubated with different primary antibodies: rabbit polyclonal antibody against NLRP3 (1:2000, Affinity, Liyang, China) or rabbit polyclonal antibody against Caspase 1 (1:2000, Affinity, Liyang, China). Incubation with the primary antibodies was performed overnight at 4 °C. For visualization, a mixture of secondary antibodies was used, goat anti-rabbit IgG conjugated with Horseradish Peroxidase (HRP) (1:500, Servicebio, Wuhan, China), with a 2 h incubation at room temperature. Subsequently, the corresponding iF488-Tyramide dye was introduced and the sample was incubated at room temperature in a light-protected chamber for a duration of 10 min. This was followed by a microwave treatment. After that, the second primary antibody, rabbit polyclonal anti-f4/80 antibody (1:500, Servicebio, Wuhan, China) was performed overnight at 4 °C, and goat anti-rabbit IgG conjugated with CY3 (1:300, Servicebio, Wuhan, China), with a 2 h incubation at room temperature. And the corresponding CY3-Tyramide dye was used as above. A ten-minute staining with DAPI was then performed at room temperature on the sections. Immunofluorescence slide scans were performed using a tissue section digital scanner (Pannoramic MIDI, 3DHISTECH, Budapest, Hungary). In this case, DAPI had an excitation wavelength of 330–380 nm, emitting blue light; 488 had an excitation wavelength of 465–495 nm, emitting green light; and CY3 had an excitation wavelength of 510–560 nm, emitting red light.

### 4.11. Isolation of RNA and Quantitative Real-Time PCR (qRT-PCR)

The TRIzol reagent (Takara Bio, Shiga, Japan) was used to extract total RNA from lung tissues and cells. Utilizing a NanoDrop spectrophotometer (Thermo Fisher, MA, USA), the absorbance at 260/280 nm was measured to determine the purity and quantity of RNA. Utilizing a cDNA synthesis kit (Monad Bio, Suzhou, China), complementary DNA (cDNA) was synthesized from the extracted RNA. We used a LightCycler 480 (Roche Molecular Systems, Inc., Basel, Switzerland) to amplify target genes, as well as SYBR PrimeScript RT-PCR kits (Monad Bio, Suzhou, China). Using β-actin as the reference gene, the relative mRNA fold changes were calculated using the 2^-ΔΔct^ method. The data presented are the average of triplicate experiments.

### 4.12. Western Blot

In order to homogenize lung samples and cultured cells, cold RIPA buffer was diluted with protease inhibitors and phenylmethanesulfonylfluoride (PMSF) (Servicebio, Wuhan, China). Enhanced BCA protein assay kits (Servicebio, Wuhan, China) were used to measure protein concentrations. Protein samples were electrophoresed on 12% SDS polyacrylamide gels and transferred to polyvinylidene fluoride membranes, according to the manufacturer’s instructions. After blocking with 5% skimmed milk, the membrane was incubated overnight at 4 °C with primary antibodies including NLRP3, Caspases-1, and β-actin (1:1000, Affinity, Liyang, China). When the membrane was washed, a suitable secondary antibody conjugated with horseradish peroxidase (1:5000, Affinity, Liyang, China) was applied and incubated at room temperature for 2 h. Finally, chemiluminescent signals were detected using a chemiluminescence imaging system ChemiDoc MP (Bio-Rad, Hercules, CA, USA), and the gray values were quantified using Image J software.

### 4.13. Statistical Analysis

A Shapiro–Wilk test was used to determine whether the data were normally distributed. Normally distributed data are presented as mean ±SEM. Multiple group comparisons were analyzed using analysis of variance (ANOVA) followed by Tukey’s post hoc test and survival rates were analyzed using the log-rank test. *p* < 0.05 was considered statistically significant. GraphPad Prism 8.3.0 software (GraphPad Software Inc., San Diego, CA, USA) was used for statistical analysis.

## 5. Conclusions

Recent evidence suggests that GDF3 exhibits pleiotropic properties, displaying both pro-inflammatory and anti-inflammatory functions. However, the role of GDF3 in sepsis-induced ALI remains largely unexplored. Therefore, our study highlighted the significant improvement in lung inflammatory damage and suppression of pro-inflammatory cytokine release in mice with ALI through GDF3. This alleviates sepsis-associated ALI by inhibiting CLP-induced macrophage pyroptosis. Consequently, in ALI and other organ injuries caused by sepsis, GDF3 may be a promising therapeutic target. Nevertheless, further research is required to investigate the pathways through which GDF3 regulates macrophage pyroptosis.

## Data Availability

Data is contained within the article.
